# 24-Epibrassinolide Enhanced Plant Antioxidant System and Cadmium Bioavailability Under Soil Cadmium Stress

**DOI:** 10.3390/plants14050765

**Published:** 2025-03-02

**Authors:** Wenle Song, Hongen Li, Ziyi Zhao, Rongrong Si, Wen Deng, Mengqi Wang, Yepu Li

**Affiliations:** International Joint Laboratory for Watershed Ecological Security in the Water Source Area of the Middle Route of South-to-North Water Diversionin Henan Province, College of Water Resources and Modern Agriculture, Nanyang Normal University, Nanyang 473061, China; 13027521127@163.com (W.S.); 16638496423@163.com (H.L.); 13137510921@163.com (Z.Z.); 15518577687@163.com (R.S.); 17816643376@163.com (W.D.); 13673475258@163.com (M.W.)

**Keywords:** phytoremediation of Cd, plant physiology and biochemistry, rhizosphere soil, fraction, bioaccumulation factor

## Abstract

Soil cadmium pollution poses significant environmental risks, prompting global concern. Previous studies have demonstrated that 24-epibrassinolide (Brs) can enhance plant photosynthesis, thereby potentially improving the efficiency of soil cadmium remediation by increasing biomass. Therefore, this study investigated the use of Brs to enhance Cd remediation by willow and alfalfa. After four months, we analyzed soil physicochemical properties, plant physiological and biochemical responses, biomass, Cd fractionation, plant Cd concentrations, and bioaccumulation factor (BCF). Willow and alfalfa cultivation without Brs increased soil pH and carbonates, reduced the exchangeable Cd fractionation, and increased Cd bound to Fe-Mn oxides and organic matter (*p* < 0.05). Conversely, Brs application increased soil total acids, increasing the bioavailable Cd (*p* < 0.05). Willow grown for four months accumulated Cd in leaves, stems, and roots at concentrations of 141.83−242.75, 45.91−89.66, and 26.73−45.68 mg kg^−1^, respectively, with leaf BCF ranging from 14.53 to 24.88. After five months, leaves of willow planted in Cd-contaminated soil (9.65 mg kg^−1^) contained 187.90−511.23 mg kg^−1^ Cd, with BCFs of 19.25−52.38. Brs also increases plant biomass by improving photosynthesis, detoxification, and antioxidant defenses. Treatments with Brs and willow extracted 1.57−1.81 times more Cd (0.56−1.37 mg pot^−1^) than without Brs (0.31−0.87 mg pot^−1^). This study offers guidelines for Cd phytoremediation and highlights an effective strategy to enhance Cd accumulation.

## 1. Introduction

Cadmium (Cd) contamination in paddy soils primarily stems from anthropogenic activities, such as industrial emissions, transportation, and agricultural practices (e.g., wastewater irrigation, pesticide, and fertilizer application), resulting in the persistent introduction of heavy metals into these soils [1]. The accumulation of Cd in paddy soils and its subsequent adverse effects on the ecosphere constitute a pervasive environmental issue that has garnered extensive global attention [2]. Various methods for removing Cd from contaminated soil exist, including physical, biological, and chemical approaches. Among these, phytoremediation stands out as a green, eco-friendly, and cost-effective technology that has garnered significant interest [3]. The utilization of hyperaccumulators in contaminated soil represents the cornerstone of phytoremediation technology [4].

Multiple non-hyperaccumulators and hyperaccumulators have been successfully employed for the remediation of Cd-contaminated soil, including *Ricinus communis* L., *Brassica juncea* L., *Festuca arundinacea*, *Stellaria media*, *Malachium aquaticum*, and *Galium aparine* [5]. Dai et al., in the study of 2023, reported that the maximum Cd contents in the Cd hyperaccumulator *Solanum nigrum* L. were 301–346 mg kg^−1^ in the root and 318–387 mg kg^−1^ in the shoot [6]. *Phytolacca acinosa* is also a common Cd-accumulating plant, and the enrichment concentration in its above-ground parts can reach 14–30 mg kg^−1^ [7]. However, the majority of these plants utilized for soil remediation are herbaceous, possessing drawbacks such as low biomass and limited economic value. Furthermore, the subsequent disposal of these plants remains challenging, hindering the sustainability of soil remediation efforts. Consequently, research has focused on the use of economically and ornamentally valuable herbaceous and woody plants for Cd phytoremediation, including willow, alfalfa, and poplar [8,9,10]. Willow and alfalfa, particularly, are notable for their well-developed root systems, substantial biomass, rapid growth rates, and high ornamental appeal. In Li et al.’s study of 2022, alfalfa removed 72.67% of the Cd in the rhizosphere soil [11]. Willows also play an important role in pollution remediation. Research indicates that after 10 years of treatment with Salix viminalis, 21% of chromium, 30% of arsenic, 54% of cadmium, 61% of zinc, 62% of copper, 63% of lead, 87% of nickel, 53% of polychlorinated biphenyls (PCBs), and up to 73% of polycyclic aromatic hydrocarbons (PAHs) were removed from the soil [12]. These attributes have led to their widespread application in municipal greening projects and Cd phytoremediation [13,14]. Our team selected a shrub willow, willow NJU513, from over 160 willow species due to its robust ability to accumulate Cd in its leaves. Notably, when soil Cd concentration is 0.91 mg kg^−1^, the average Cd concentration in the leaves after three months is 7.99 ± 0.54 mg kg^−1^, with a corresponding bioaccumulation factor (BCF) of 8.78 [15]. However, at present, research on the effect of the willow-alfalfa intercropping system on soil cadmium remediation is still limited.

In general, the spatial arrangement of plants with varying heights in the construction of greenbelts can optimize space utilization and often enhance ornamental value. From the phytoremediation perspective, the intercropping of shrub plants and herbs facilitates the formation of root systems at different soil depths, allowing the plants to effectively absorb Cd from various soil layers and minimize remediation blind spots. Previous studies have documented the intercropping of maize with other herbs for the remediation of soil heavy metals, including cowpea, purple haricot, chickpea, alfalfa, teosinte, amaranth, and rape [16]. However, intercropping often results in reduced plant biomass compared to monoculture. A previous study reported that intercropping willow with herbaceous plants *Lolium perenne* L., *Iris lactea* Pall., and *Bidens pilosa* L. enhanced the phytoremediation efficiency by increasing the Cd accumulation in plants; however, a reduction in the biomass of the herbaceous plants was also observed [17]. A similar result was indicated by the intercropping of tomato with *Sedum alfredii* in the Cd-contaminated soil [18]. In our prior research, urea was employed to augment plant biomass and tissue Cd concentrations [9]. Brs, a class of polyhydroxysteroids involved in diverse physiological processes and morphological responses in plants, have been shown to mitigate the effects of abiotic stresses, such as heavy metals, by activating various mechanisms within plants, including enhancing the activity of enzymatic antioxidants like superoxide dismutase (SOD), catalase (CAT), and glutathione reductase [19]. After the application of Brs, the biomass of two canola varieties (*Brassica napus* L.) increased under salt-stress conditions [20], and their photosynthetic activity was also enhanced. Consequently, Brs were utilized to counteract soil Cd stress and increase plant biomass. The total concentration of heavy metals in soil often fails to accurately reflect their bioavailability to plants [14]. According to Tessier’s research conducted in 1979, the total concentration of Cd in the soil can be fractionated into exchangeable (EX-Cd), carbonate-bound (CAB-Cd), Fe-Mn oxide-bound (FMO-Cd), organic-bound (OM-Cd), and residual fractions (RES-Cd) [21]. Among these fractions, exchangeable fractionation is the bioavailable fractionation that plants can readily utilize. The carbonate-bound, Fe-Mn oxide-bound, and organic-bound fractions constitute the potentially bioavailable fractionation, which can be released and absorbed by plants under altered environmental conditions. Conversely, the residual fractionation, being encapsulated within mineral lattices, is not accessible for plant uptake. Multiple studies have indicated that the bioavailability of heavy metals is influenced by the soil’s physicochemical properties, such as pH, oxidation-reduction potential (OPR), electrical conductivity (EC), carbonates (CAB), organic matter (OM), and organic acid [22,23,24,25]. Furthermore, there is a paucity of information regarding the changes of Cd in soil and plants following Brs treatment.

The existing studies on soil Cd phytoremediation are insufficient, demonstrating limited efficiency in Cd removal, low plant biomass, and insufficient resistance to Cd stress. Furthermore, scant information is available on the enhancement of phytoremediation ability by alfalfa willow through the application of Brs. Therefore, the objectives of the current study were to (1) investigate the phytoremediation potential of willow NJU513 in Cd-contaminated soils; (2) evaluate the impact of Brs on the phytoremediation effectiveness of willow, alfalfa, and their intercropping combination; and (3) explore the fractionation of Cd in soil treated with and without Brs.

## 2. Materials and Methods

### 2.1. Pot Culture Experiment

The experimental soil with Cd pollution, collected from the agricultural land situated in Yixing City, Jiangsu Province, China (longitude: 119.6936° E, latitude: 31.4069° N), was derived from the upper 0−20 cm layer of oozing paddy soil. Prior to being added into pots, the soil was processed by removing stones and plant residues, followed by natural drying and grinding to ensure particle sizes were less than 2 mm. The physicochemical properties of the control soil (CK) and Cd-polluted soil were recorded in Table 1.

This study involved the following three distinct treatment groups: a single willow (NJU513, *S. babylonica × S. × Leucopithecia* ‘NJU513′) treatment, a single alfalfa (*Medicago sativa* L.) treatment, and an intercropping treatment combining willow and alfalfa. The specific configurations and corresponding numbers of each treatment are outlined in Table 2. A total of 1 kg of Cd-polluted soil was utilized in plastic pots. For the low Cd-contaminated control soils (CK), willow (CK1), alfalfa (CK2), and their intercrop (CK3) were planted. In Cd-contaminated soils, willow (W-NBrs), alfalfa (A-NBrs), and their intercrop (WA-NBrs) were planted. Additionally, willow (W-Brs), alfalfa (A-Brs), and their intercrop (WA-Brs) in Cd-contaminated soils were treated with Brs spray every seven days. The pots with Cd-contaminated soil but without any planting of willow or alfalfa were designated as NWABrs. Each treatment was replicated four times. Willow cuttings, approximately 1 cm in diameter and 5 cm in length were sourced from the experimental base of Nanjing University in Baguazhou, Nanjing, China. Alfalfa seeds were acquired from Jiangsu Yaju Seed Industry, with 1 g of seeds sowed per pot and thinned to four plants per pot after 15 days. The uniform cuttings were rooted and grown in seedling boxes filled with Hoagland solution for four weeks in a greenhouse, as per Paganeli et al. [26]. Subsequently, uniform saplings were selected and planted in the greenhouse maintained at a temperature range of 23 to 28 °C and a relative humidity of 60 to 65%. After four months of growth (Figure S1), the willow and alfalfa plants were harvested and separated into roots, stems, and leaves. The tissues were thoroughly rinsed with deionized water, freeze-dried to a consistent weight, and analyzed. Rhizosphere soil was collected as described by Yu et al. [27], and unless otherwise specified, all soils mentioned in this study refer to rhizosphere soils.

### 2.2. Plants and Soils Analysis

#### 2.2.1. Soil Properties

The soil pH, OPR, and EC were measured using clarified liquor in a 1:2.5 ratio with the aid of an instrument manufactured by OHAUS (Starter 3100, Parsippany, NJ, USA). Soil organic matter (SOM) was quantified through the potassium dichromate oxidation method, while CAB was assessed using gas volume techniques, as described by Lu [28]. Additionally, soil total organic acidity (TOA) was determined by a colorimetric method in accordance with the procedures outlined by Liu et al. [29].

#### 2.2.2. Determination of Cd in Soil and Plants

One gram of soil that passed through a 100-mesh sieve was used for the extraction of Cd fractionation in the soil. The fractionation of Cd in soil was analyzed using Tessier’s sequential extraction procedure [21]. Soil Cd was extracted by 1 mol L^−1^ MgCl_2_·6H_2_O in the EX-Cd; by 1 mol L^−1^ HAc-NaAc in the CAB-Cd; by 0.04 mol L^−1^ HONH_2_HCl in the bound to FMO-Cd; by HNO_3_ (0.02 mol L^−1^)-H_2_O_2_ (30%)-NH_4_Ac (3.2 mol L^−1^) in the bound to OM-Cd; and by HNO_3_-HCl-HF-H_2_O_2_ digestion in the total Cd and bound to RES-Cd. A total of 0.3 g and 0.5 g of air-dried soil and plant tissues that have passed through a 100-mesh sieve were used for the digestion of total Cd in the soil and plant, respectively. The concentrations of Cd in plant tissues and soil were digested using a 1:3 ratio mixture (HNO_3_:HCl) in a graphite digester (Watenv, YS50-48, Shanghai, Chnia) following the protocol established by Ajah et al. and Aliyeva et al. [30,31]. For the digestion of plants, the temperature was maintained at 100 °C for 1 h, followed by 3 h at 200 °C. For the digestion of soils, the temperature was maintained at 100 °C for 3 h, followed by 4 h at 200 °C. The suspension was obtained from the digestion and filtration, which was then reconstituted to a desired volume (50 mL) and analyzed by an atomic absorption spectrophotometer (HITACHI, Z2000, Chiyoda City, Japan). The certified reference samples GSS-8 and GSB-6 (purchased from the Institute of Geophysical and Geochemical Exploration, Chinese Academy of Geological Sciences) were used to ensure the recovery (100% ± 5) of Cd in the soil and plant samples. The concentrations of Cd in the working curve are 0 mg L^−1^, 0.1 mg L^−1^, 0.2 mg L^−1^, 0.5 mg L^−1^, 1 mg L^−1^, 2 mg L^−1^, and 5 mg L^−1^, and the detection limit is 10^−3^ mg L^−1^.

#### 2.2.3. Determination of Plant Physiology and Biochemistry

A total of 0.2 g of mature plant leaves were utilized for the determination of photosynthetic pigments and the assessment of the antioxidant defense system. The concentrations of catalase (CAT), total superoxide dismutase (T-SOD), malondialdehyde (MDA), and total thiols (T-SH) in the plant leaves were measured using kits provided by Nanjing Jiancheng Bioengineering Institute (http://www.njjcbio.com). The photosynthetic pigments were extracted with acetone, followed by spectrophotometric determination of chlorophyll *a* (*Chl a*) at 663.2 nm, chlorophyll *b* (*Chl b*) at 646.8 nm, and carotenoids at 470.0 nm using a UV-1800 spectrophotometer (MAPADA, Shanghai, China), as per the method described by Cambrollé et al. [32]. The net photosynthesis (*P_n_*) was measured using a Li-6800 portable photosynthesis system from LiCor Inc. (Lincoln, NE, USA). The photosynthetic parameters, including light intensity (1000 μmol photon m^−2^ s^−1^), airflow (700 μmol s^−1^), and CO_2_ concentration (400 μmol mol^−1^), were established based on the guidelines provided by Li et al. [9]. The detection ranges of T-SOD, CAT, MDA, GSH, *Chl a*, *Chl b*, and carotenoids (in terms of concentration in liquid medium) are 0.5–122.1 U mL^−1^, 0.2–24.8 U mL^−1^, 0.5–113.0 nmol mL^−1^, 20–330 μmol mL^−1^, 0.01–5 mg L^−1^, 0.005–2 mg L^−1^, and 0.002–1 mg L^−1^, respectively.

#### 2.2.4. Determination of Bioaccumulation Factor in Plants

To assess the accumulation capacity of plants for cadmium (Cd), the BCF is calculated using the following equation, as described by Zhang et al. [33]:

BCF_root/stem/leaves_ = Cd_root/stem/leaves_/Cd_soil_

### 2.3. Data Analysis

The average values, along with their corresponding Figures and standard deviations (SD), were computed using Excel (Office 2019 for Windows). The Pearson correlation coefficient (2-tailed) was obtained using SPSS version 22.0. Different letters in the Figures denote significant differences among various treatments based on the least square difference method (Duncan, *p* < 0.05). Significance levels are indicated by asterisks; * denotes a significant difference at the *p* < 0.05 level, while ** denotes a significant difference at the *p* < 0.01 level.

## 3. Results

### 3.1. Soil Physicochemical Properties

The physicochemical properties of the soil (pH, ORP, OM, EC, TOA, and CAB) were analyzed and presented in Table 3. After four months, across all treatments, soil ORP, OM, and TOA significantly decreased compared to NWABrs (*p* < 0.05), while soil EC and CAB were higher, with EC showing a significant increase (*p* < 0.05). Moreover, soil pH in W-NBrs, A-NBrs, and WA-NBrs treatments was significantly higher than in NWABrs (*p* < 0.05). Comparing W-Brs, A-Brs, and WA-Brs with W-NBrs, A-NBrs, and WA-NBrs, the application of Brs reduced soil pH and ORP but increased soil TOA.

### 3.2. Plant Physiology and Biochemistry Responses

**Figure 1:**
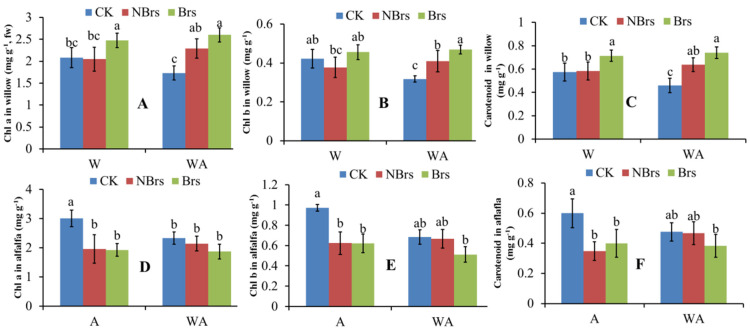
*Chl a*, *Chl b*, and carotenoid in willow and alfalfa treated with and without Brs after four months. (**A**–**C**) indicate *Chl a*, *Chl b*, and Carotenoid for willow, respectively. (**D**–**F**) indicate *Chl a*, *Chl b*, and Carotenoid for alfalfa, respectively. W, A, and WA indicate that soils were planted with willow, alfalfa, and a combination of them, respectively. CK indicates that willow, alfalfa, and a combination of them were planted in the soil with 0.67 mg kg^−1^ Cd pollution. Brs and NBrs indicate that willow, alfalfa, and a combination of them were treated with and without Brs in the Cd-polluted soils (9.65 mg kg^−1^), respectively. The different letters indicate the significant differences among different treatments.

**Figure 2:**
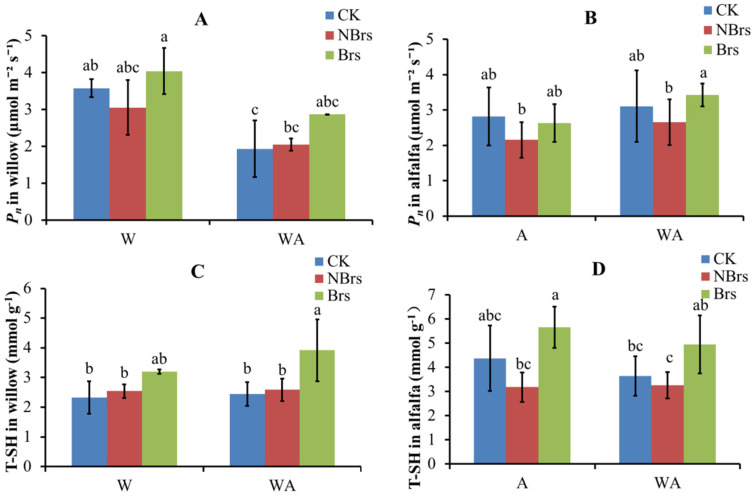
*P_n_* and T-SH in willow and alfalfa treated with and without Brs after four months. (**A**,**B**) indicate *P_n_* for willow and alfalfa, respectively. (**C**,**D**) indicate T-SH for willow and alfalfa, respectively. W, A, and WA indicate that soils were planted with willow, alfalfa, and a combination of them, respectively. CK indicates that willow, alfalfa, and a combination of them were planted in the soil with 0.67 mg kg^−1^ Cd pollution. Brs and NBrs indicate that willow, alfalfa, and a combination of them were treated with and without Brs in the Cd-polluted soils (9.65 mg kg^−1^), respectively. The different letters indicate the significant differences among different treatments.

As shown in Figure 1A–C, compared with the CK, soil Cd did not harm the photosynthetic pigment concentrations in willow leaves in W-NBrs and WA-NBrs treatments. In contrast, the photosynthetic pigment concentrations in W-Brs and WA-Brs treatments were higher than those in W-NBrs and WA-NBrs. Similarly, the trends of *P_n_* and T-SH in willow leaves resembled those of photosynthetic pigments (Figure 2A,C). Unlike in willow, soil Cd negatively affected the photosynthetic pigment concentrations in alfalfa compared to the CK, and Brs application did not increase these concentrations (Figure 1D–F). Compared to the CK, soil Cd reduced the T-SH and *P_n_* of alfalfa leaves in A-NBrs and WA-NBrs treatments. However, the T-SH and *P_n_* of alfalfa in A-Brs and WA-Brs were significantly higher than in the corresponding A-NBrs and WA-NBrs treatments and did not significantly differ from the CK (Figure 2B,D).

**Figure 3:**
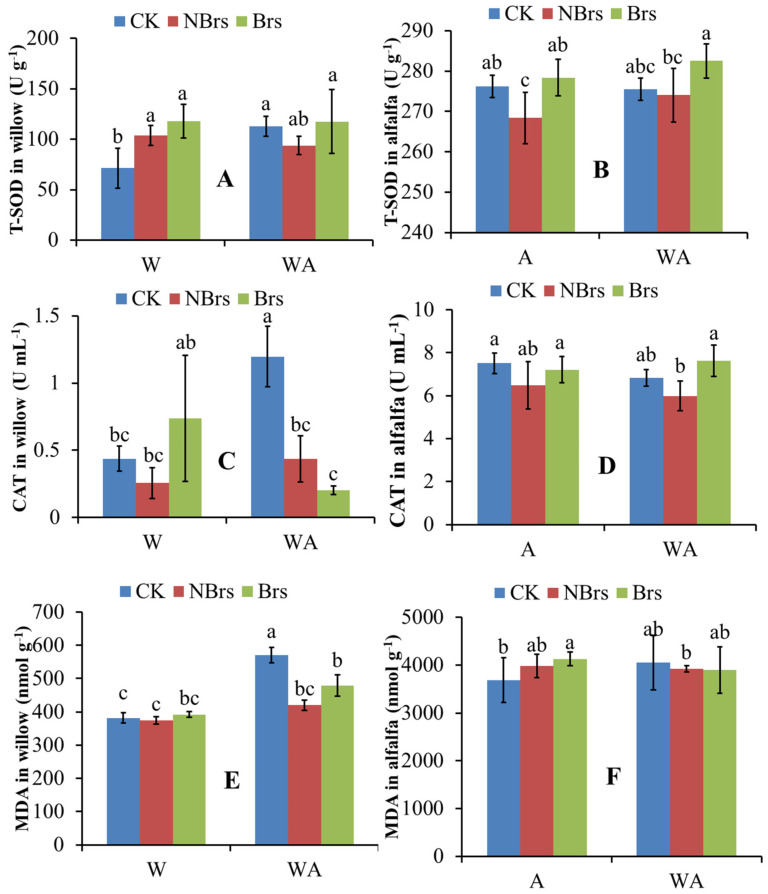
T-SOD, CAT, and MDA in willow and alfalfa were treated with and without Brs after four months. (**A**,**C**,**E**) indicate T-SOD, CAT, and MDA for willow, respectively. (**B**,**D**,**F**) indicate T-SOD, CAT, and MDA for alfalfa, respectively. W, A, and WA indicate that soils were planted with willow, alfalfa, and a combination of them, respectively. CK indicates that willow, alfalfa, and a combination of them were planted in the soil with 0.67 mg kg^−1^ Cd pollution. Brs and NBrs indicate that willow, alfalfa, and a combination of them were treated with and without Brs in the Cd-polluted soils (9.65 mg kg^−1^), respectively. The different letters indicate the significant differences among different treatments.

Figure 3 shows the activities of T-SOD, CAT, and MDA in willow and alfalfa leaves. Generally, monocropping and intercropping treatments had little significant impact on T-SOD, CAT, and MDA in both plants. In alfalfa, compared with the CK, soil Cd in A-NBrs and WA-NBrs treatments reduced T-SOD and CAT activities, while MDA remained unchanged (Figure 3B,D,F). However, Brs application in A-Brs and WA-Brs increased T-SOD and CAT activities without significantly altering MDA compared to A-NBrs and WA-NBrs (Figure 3B,D,F).

In willow, compared with the CK, soil Cd in W-NBrs did not significantly reduce T-SOD and CAT activities, and Brs application enhanced them (Figure 3A,C). As a result, MDA concentrations did not differ significantly among the control, W-NBrs, and W-Brs (Figure 3E). In the willow intercropping treatment, soil Cd had no significant effect on T-SOD compared to the control. Although soil Cd significantly suppressed CAT activity (*p* < 0.05), MDA concentrations were significantly decreased.

### 3.3. Plant Biomass

**Figure 4:**
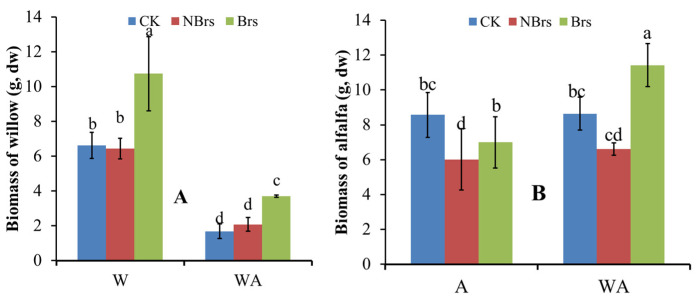
Plant total biomass of willow (**A**) and alfalfa (**B**) treated with and without Brs after four months. W, A, and WA indicate that soils were planted with willow, alfalfa, and a combination of them, respectively. CK indicates that willow, alfalfa, and a combination of them were planted in the soil with 0.67 mg kg^−1^ Cd pollution. Brs and NBrs indicate that willow, alfalfa, and a combination of them were treated with and without Brs in the Cd-polluted soils (9.65 mg kg^−1^), respectively. The different letters indicate the significant differences among different treatments.

Figure 4A,B, respectively, show the total production of willow and alfalfa after four months of growth. Compared with the CK, soil Cd had no negative effect on the total biomass of willow in W and WA treatments. When Brs was sprayed, the average biomass per pot was 10.74 g in W-Brs and 3.71 g in WA-Brs, 1.62-fold and 2.21-fold higher than the CK in monocrop and intercrop treatments, respectively (Figure 4A).

In contrast to willow, soil Cd reduced the total biomass of alfalfa in A-NBrs and WA-NBrs treatments by 29.84% and 23.43%, respectively, compared to the CK. However, Brs spray significantly increased the total biomass by 14.03% and 42.10%, respectively, compared to A-NBrs and WA-NBrs (*p* < 0.05; Figure 4B). Notably, alfalfa in the WA-Brs treatment had the highest total biomass among all treatments in Figure 4B.

### 3.4. Fractionation of Cd in Soil

**Figure 5:**
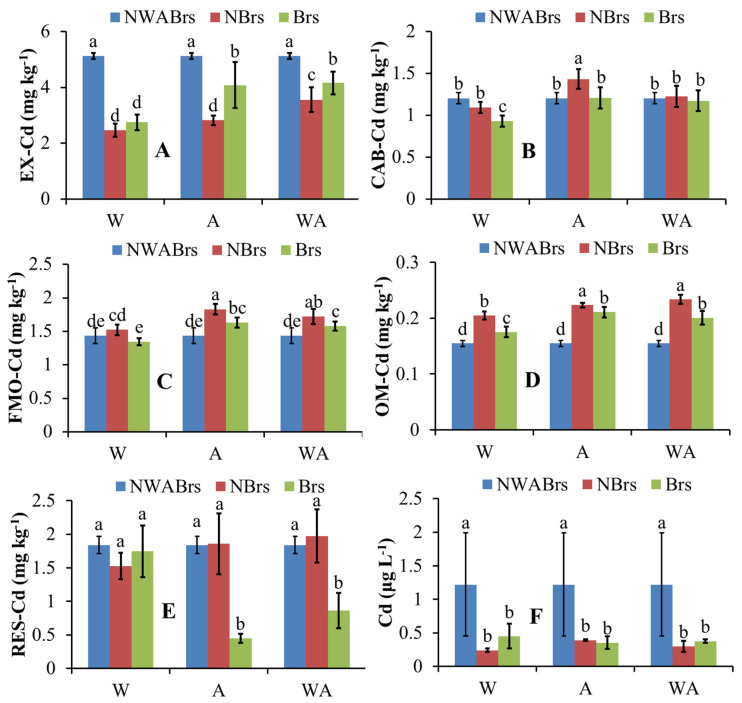
Concentrations of EX-Cd (**A**), CAB-Cd (**B**), FMO-Cd (**C**), OM-Cd (**D**), RES-Cd (**E**), and ion Cd (**F**) in the rhizosphere soil after plant four months of growth. W, A, and WA indicate that soils were planted with willow, alfalfa, and a combination of them, respectively. Brs and NBrs indicate that willow, alfalfa, and a combination of them were treated with and without Brs in the Cd-polluted soils (9.65 mg kg^−1^), respectively. The different letters indicate the significant differences among different treatments.

After four months of plant growth, the fractionation of soil Cd (EX-Cd, CAB-Cd, FMO-Cd, OM-Cd, RES-Cd, and ionic Cd) was extracted and shown in Figure 5. In the original soil (NWABrs), EX-Cd was the primary fraction, accounting for 52.54% of total Cd, followed by RES-Cd, FMO-Cd, CAB-Cd, and OM-Cd.

Planting willow, alfalfa, or their combination significantly reduced EX-Cd concentrations compared to NWABrs (*p* < 0.05). Specifically, in W-NBrs, A-NBrs, WA-NBrs, W-Brs, A-Brs, and WA-Brs, EX-Cd concentrations were 48.08%, 55.12%, 69.30%, 53.63%, 79.64%, and 81.13% of the initial soil (NWABrs) concentrations, respectively. Conversely, OM-Cd concentrations in soils with willow, alfalfa, or both were significantly higher than in NWABrs (*p* < 0.05). Brs spray significantly decreased OM-Cd compared to W-NBrs, A-NBrs, and WA-NBrs, a similar trend was observed for FMO-Cd (*p* < 0.05). However, FMO-Cd concentrations in W-NBrs, A-NBrs, WA-NBrs, A-Brs, and WA-Brs were significantly higher than in NWABrs (*p* < 0.05).

Among all treatments, CAB-Cd concentrations showed no significant differences, except in W-Brs (significantly lower) and A-NBrs (significantly higher) than NWABrs (*p* < 0.05). For RES-Cd, there were no significant changes across treatments, except in A-Brs and WA-Brs, where concentrations were 24.38% and 46.80% of the initial soil, respectively.

The concentration of Cd extracted by deionized water in the initial soil was 1.22 μg L^−1^, significantly higher than in soils with willow and alfalfa (0.24–0.45 μg L^−1^; Figure 5F, *p* < 0.05).

### 3.5. Concentrations of Cd in Willow and Alfalfa

**Figure 6:**
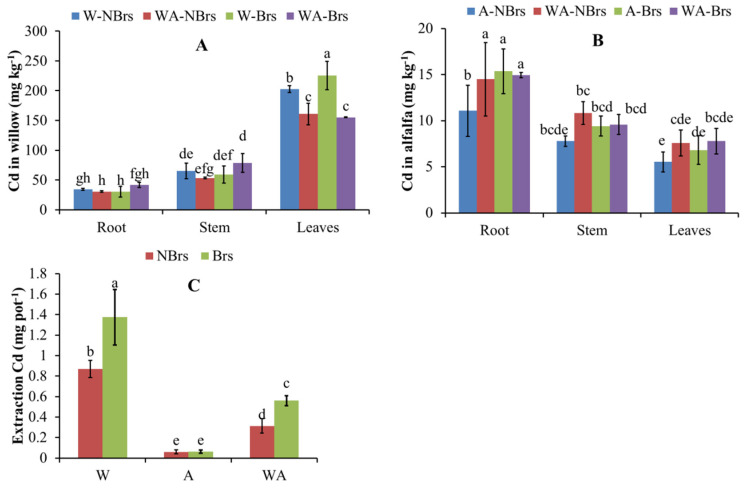
Concentrations of Cd in the plant tissues ((**A**) for willow and (**B**) for alfalfa) and the total extraction contents (**C**) by plants after four months of growth. W, A, and WA indicate that soils were planted with willow, alfalfa, and a combination of them, respectively. Brs and NBrs indicate that willow, alfalfa, and a combination of them were treated with and without Brs in the Cd-polluted soils (9.65 mg kg^−1^), respectively. The different letters indicate the significant differences among different treatments.

Figure 6 shows the Cd concentrations in the plant tissues of willow and alfalfa. In all treatments, in willow, Cd concentrations were highest in leaves (141.83–242.75 mg kg^−1^), followed by stems (45.91–89.66 mg kg^−1^) and roots (26.73–45.68 mg kg^−1^; Figure 6A). In contrast, in alfalfa, the order was roots (11.08–15.37 mg kg^−1^) > stems (7.78–10.82 mg kg^−1^) > leaves (5.54–7.78 mg kg^−1^).

Brs spray significantly increased Cd concentrations in the stems of WA-Brs and the leaves of W-Brs compared to their non-Brs counterparts (*p* < 0.05), but there were no significant changes in other plant parts (Figure 6A). In alfalfa, Brs spray significantly increased Cd concentrations in the roots of A-Brs relative to A-NBrs. There were no significant differences in Cd concentrations between WA-NBrs and WA-Brs for roots, between A-NBrs/WA-NBrs and A-Brs/WA-Brs for stems, and between A-NBrs/WA-NBrs and A-Brs/WA-Brs for leaves.

The average extraction contents of Cd in W-NBrs, W-Brs, A-NBrs, A-Brs, WA-NBrs, and WA-Brs were 0.87, 0.06, 0.31, 1.37, 0.06, and 0.56 mg pot^−1^, respectively (Figure 6C). Notably, the extraction contents of Cd in A-Brs and WA-Brs were 1.57 and 1.81 times higher than those in A-NBrs and WA-NBrs, respectively.

### 3.6. Bioaccumulation Factor of Cd in Willow and Alfalfa

**Figure 7:**
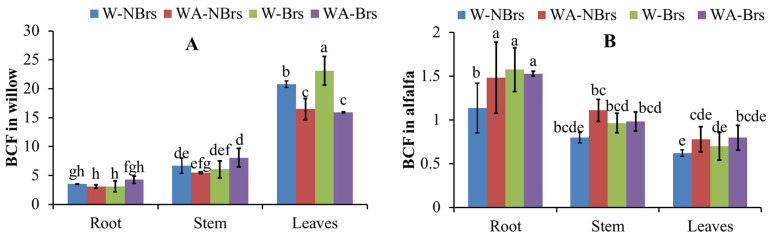
Bioaccumulation factor of Cd in the different tissues of willow (**A**) and alfalfa (**B**). W, A, and WA indicate that soils were planted with willow, alfalfa, and a combination of them, respectively. Brs and NBrs indicate that willow, alfalfa, and a combination of them were treated with and without Brs in the Cd-polluted soils (9.65 mg kg^−1^), respectively. The different letters indicate the significant differences among different treatments.

The BCF of Cd in the plant tissues of willow and alfalfa are presented in Figure 7. For willow, the BCF for roots stems, and leaves ranged from 2.39 to 4.80, 4.70 to 9.19, and 14.53 to 24.88, respectively. Notably, the application of Brs spray significantly increased the BCF in the stems of WA-Brs and the leaves of A-Brs compared to their respective controls, WA-NBrs for stems and A-NBrs for leaves (*p* < 0.05). In contrast, for alfalfa, the BCF for roots stems, and leaves ranged from 0.89 to 1.95, 0.75 to 1.29, and 0.41 to 0.96, respectively, across all treatments. These values were lower than those observed in willow. Additionally, the application of Brs spray significantly increased the BCF in the roots of A-Brs compared to A-NBrs (*p* < 0.05).

## 4. Discussion

### 4.1. Plant Biomass

Plant biomass is a pivotal factor influencing soil Cd phytoremediation [34]. When compared to the CK for monoculture, soil Cd did not significantly impact the total plant biomass of willow in the absence of Brs spray (W-NBrs), whereas Brs significantly enhanced the biomass in W-Brs (Figure 4A, *p* < 0.05). These research findings agreed with the alterations observed in the photosynthetic rate (*P_n_*) (as depicted in Figure 2A), and this was consistent with the study conducted by Pilipović et al. [35]. Photosynthetic pigments have been shown to be positively correlated with *P_n_* in plants [36,37]. In the present study, soil Cd did not significantly alter the concentrations of photosynthetic pigments (chlorophyll *a*, chlorophyll *b*, and carotenoid) in W-NBrs compared to the CK. Conversely, Brs spray improved the photosynthetic pigments in W-Brs relative to the CK, which was also indicated in a previous study [38]. Therefore, these variations in photosynthetic pigments can be recommended as the direct cause for the changes in total plant biomass in W-NBrs and W-Brs [37]. CAT and SOD enzymes play crucial roles in plant growth, including defense against oxidative damage, regulation of antioxidant enzymes, mutual coordination between classes, and elimination of intracellular reactive oxygen species, thereby preventing cell membrane peroxidation [39]. Heavy metal ions in the cytosol often bind to chelators with sulfhydryl groups, such as glutathione and phytochelatins, to alleviate heavy metal toxicity in plants [40]. In this study, soil Cd did not significantly affect T-SOD, CAT, and T-SH content in W-NBrs compared to the CK for monoculture, while Brs spray increased their levels in W-Brs compared to both the CK and W-NBrs (Figure 3A,C,E). MDA is an indicator of the degree of cell damage following Cd stress, and its value increases with the increase in the degree of cell damage [41]. In the current research, compared with the control group (CK), the MDA concentrations in the W-NBrs and W-Brs groups did not show statistically significant changes. This finding indicated that the willow variety NJU513 demonstrates a robust capacity to resist Cd stress. Therefore, the changes in T-SOD, CAT, and T-SH in W-NBrs and W-Brs can be considered as another reason why plant biomass was not negatively affected by Cd (Figure 4A).

Similar to W-NBrs and W-Brs, the total plant biomass of willow in the absence of Brs spray in intercropped conditions (WA-NBrs) was not significantly affected by soil Cd compared to the CK for intercrop, and Brs spray significantly increased plant total biomass compared to WA-Brs (Figure 4A). The changes in photosynthetic pigments and *P_n_* can be used to explain the phenomenon observed in WA-NBrs and WA-Brs, as mentioned for W-NBrs and W-Brs. According to Figure 4A–C, the average concentrations of photosynthetic pigments in WA-NBrs and WA-Brs were higher than those in W-NBrs and W-Brs, respectively. However, the average *P_n_* in WA-NBrs and WA-Brs was lower than in W-NBrs and W-Brs, respectively, resulting in a significant decrease in willow total biomass in the intercropped conditions compared to monoculture (Figure 2A and Figure 4A, *p* < 0.05). This phenomenon between monoculture and intercrop suggests that intercropping disrupts the structure of the chlorophyll photosystem II due to limited living space, reducing the photosynthetic performance of leaves [42]. In this study, the average values of T-SOD in WA-NBrs and CAT in WA-Brs were lower than those in W-NBrs and W-Brs, respectively, followed by an increase in MDA in WA-NBrs and WA-Brs, which may disrupt the membrane system for photosynthesis (Figure 3A,C,E) [43]. These results indicated that the intercropping system of alfalfa and willow exhibited greater tolerance compared to other intercropping combinations, such as rice-*Amaranthus* and rice-*Perilla* [44]. This could be attributed to the fact that the decrease in biomass in the alfalfa-willow intercropping system was less significant than that in the individual cropping of these species.

Soil Cd decreased the average values of *P_n_* in alfalfa in the absence of Brs spray (A-NBrs) and intercropped conditions (WA-NBrs) compared to the CK for monoculture and intercrop (Figure 2B). Liu et al. also indicated that with the increase in Cd concentration, *P_n_* of alfalfa decreased gradually [45]. These results can be used to explain the changes in alfalfa total biomass shown in Figure 4B. From the perspective of plant physiology and biochemistry, photosynthetic pigments, T-SH, T-SOD, and CAT in A-NBrs and WA-NBrs were lower than the CK, indicating that soil Cd suppresses antioxidant, photosynthesis, and detoxification abilities [46], resulting in suppressed alfalfa total biomass in A-Brs and WA-Brs [47]. Photosynthetic pigments in A-Brs and WA-Brs were lower than those in the CK for monoculture and intercrop, but the *P_n_* values were higher than the CK. The previous study has indicated that the concentration of Brs at 0.208 μmol L^−1^ under salt stress significantly increased the net photosynthetic rate [48]. These results suggest that Brs spray enhanced the photosynthetic property of chlorophyll and thus improved plant total biomass in A-Brs and WA-Brs. Additionally, Brs spray increased the average values of T-SH, T-SOD, and CAT in A-Brs and WA-Brs compared to the CK, with MDA not being affected by soil Cd, contributing to the increase in alfalfa total biomass in A-Brs and WA-Brs.

### 4.2. Fractionation of Cd in Soil

Generally, heavy metals present in the exchangeable fraction are considered readily and potentially bioavailable, making them crucial for assessing the risk of metal leaching into groundwater and their subsequent absorption by plants [34,49]. In the current study, significant correlations were observed between the concentrations of EX-Cd and parameters such as ORP (r = 0.627 **, *p* < 0.01), EC (r = −0.422 *, *p* < 0.05), and TOA (r = 0.408 *, *p* < 0.05) (Supplemental Materials, Table S2). Shen et al. [50] reported a decrease in EX-Cd with decreasing ORP and increasing pH. Consequently, the EX-Cd concentrations in soil planted with plants were lower than those in non-planted soils (NWABrs), potentially due to changes in ORP, pH, or EC (Table 3). In treatments involving plants, the concentrations of EX-Cd and water-extracted Cd were notably lower than those in NWABrs (Figure 5A). Specifically, the concentrations of water-extracted Cd in both rhizosphere (0.24 to 0.45 μg L^−1^, Figure 5F) and non-rhizosphere (0.23 to 0.63 μg L^−1^, Supplemental Materials, Figure S2) were below China’s groundwater quality standards for the second class (1 μg L^−1^) (SAC, 2017). Additionally, the total Cd content in rhizosphere soils was lower than that in non-rhizosphere and initial soils (Supplemental Materials, Table S1). These findings collectively suggest the effectiveness and safety of the phytoremediation strategy employed in this study.

Xiang et al. [51] proposed that plants can absorb heavy metals bound to exchangeable and carbonate fractionations. Thus, the decrease in CAB-Cd in willow-planted soils (W-Brs) may be attributed to plant accumulation, given that willows accumulated the highest Cd amounts (Figure 5B and Figure 6C). Heavy metals bound to the carbonate fraction are often precipitated or co-precipitated with carbonates and are sensitive to pH changes [49]. Therefore, CAB-Cd concentrations in the A-NBrs reached the highest levels among all treatments, likely due to their highest pH and carbonate content (Table 3). In treatments without Brs, EX-Cd concentrations significantly decreased compared to NWABrs, whereas CAB-Cd, FMO-Cd, OM-Cd, and RES-Cd concentrations showed no significant changes or increased compared to NWABrs (*p* < 0.05, Figure 5). These results indicate that the increases in CAB-Cd, FMO-Cd, and OM-Cd originated from EX-Cd, as reported by Zhao et al. [52] stated that EX-Cd can convert into reducible and oxidizable forms under certain conditions. Plants extracted more Cd in treatments with Brs than in those without Brs (Figure 6C), yet EX-Cd concentrations were also higher in treatments with Brs (Figure 5A). These phenomena suggest that part of the EX-Cd in treatments with Brs originated from the transformation of other fractions. In contrast, CAB-Cd concentrations in both A-Brs and WA-Brs showed no significant changes compared to their corresponding treatments (Figure 5B), while FMO-Cd and OM-Cd concentrations in A-Brs and WA-Brs were significantly higher (Figure 5C,D). Therefore, part of the EX-Cd, FMO-Cd, and OM-Cd in A-Brs and WA-Brs may have originated from the decrease in RES-Cd (Figure 5E). Notably, OM-Cd concentrations were significantly negatively correlated with RES-Cd (r = −0.663 **, *p* < 0.01), providing statistical support for this hypothesis (Supplemental Materials, Table S2).

The present study also revealed that RES-Cd concentrations in A-Brs and WA-Brs were significantly lower than those in NWABrs (Figure 5E), and soil pH was positively correlated with RES-Cd (Supplemental Materials, Table S2; r = 0.497 **, *p* < 0.01). However, soil pH in this study may have been strongly influenced by TOA, given their significant negative correlation (Supplemental Materials, Table S2; r = −0.548 **, *p* < 0.01). Thus, compared to treatments without Brs, the decrease in soil pH in treatments with Brs may be attributed to increased soil TOA. Additionally, soil TOA was closely correlated with ORP (r = 0.540 **, *p* < 0.01), EC (r = −0.520 **, *p* < 0.01), CAB (r = −0.478 *, *p* < 0.05), and OM (r = 0.535 **, *p* < 0.01) due to significant correlations among these parameters. These factors (pH, TOA, ORP, EC, CAB, and OM) may indirectly or directly reduce residual Cd, although the underlying mechanisms require further investigation. Yang et al. [53] reported significant negative correlations between FMO-Cd and OM-Cd concentrations and soil pH. Therefore, the decrease in soil pH due to increased TOA can explain the lower concentrations of FMO-Cd and OM-Cd in W-Brs, A-Brs, and WA-Brs compared to without Brs (W-NBrs, A-NBrs, and WA-NBrs), respectively.

### 4.3. Phytoremediation of Cd by Willow and Alfalfa

Brs plays a pivotal role not only in plant developmental processes such as photomorphism, pollen tube growth, and root inhibition but also in enhancing plant resilience against various stressors [54]. Specifically, for willow and alfalfa, the application of Brs spray significantly elevated Cd concentrations in WA-Brs (willow stems) and W-Brs (willow leaves) in comparison to the respective treatments without Brs (Figure 6A, *p* < 0.05). Notably, the extraction efficiency of Cd in both WA-Brs and W-Brs was also markedly increased (Figure 6C, *p* < 0.05). Plant biomass and bioavailable Cd (EX-Cd) are key factors affecting the enhancement of phytoremediation efficiency and enrichment concentration, positively correlated with Cd accumulation [55,56]. Therefore, these observations can be attributed to the Brs spray’s ability to augment plant total biomass and EX-Cd, thereby facilitating Cd accumulation in plants (Figure 4 and Figure 5A). Across all treatments, the application of Brs spray resulted in a higher total extraction of Cd by both willow and alfalfa compared to the control treatments, suggesting that the Brs strategy represents an environmentally friendly and effective method for enhancing Cd phytoremediation.

According to Furini’s definition [57], Cd hyperaccumulator plants are characterized by Cd concentrations in their leaves or aboveground parts (dry weight) exceeding 100 mg kg^−1^, with a critical toxicity level ranging from 6 to 10 mg kg^−1^ (dry weight). In the present study, after four months, the Cd concentrations in the leaves of W-NBrs ranged from 197.80 to 209.06 mg kg^−1^, with an average BCF of 20.78 (Figure 6A). An additional analysis of six randomly selected willow leaves, conducted five months later using the same soil (Cd = 9.65 mg kg^−1^, Figure S3), revealed Cd concentrations varying from 187.90 to 511.23 mg kg^−1^, with a BCF of 19.25 to 52.38 (Figure S4). Importantly, the total biomass, T-SOD, CAT, T-SH, *P_n_*, and photosynthetic pigments in W-NBrs were not significantly suppressed compared to the CK, and MDA concentrations did not significantly differ from CK. Furthermore, the BCF of stems and leaves was significantly higher than that of roots. These findings suggest that willow (NJU513) has potential as a Cd hyperaccumulator, exhibiting higher Cd phytoremediation potential than *Salix alba* (Cd concentration < 15 mg kg^−1^) [58]. Wei et al. [59] reported that *Solanum nigrum* L. is a Cd hyperaccumulator with leaf Cd concentrations of 124.60 mg kg^−1^ when soil Cd is 25 mg kg^−1^, which is lower than the Cd concentrations observed in the leaves of the willow species studied here.

The long-term effects of Brs application on soil health and the sustainability of this method are also critical factors influencing its application prospects. Brs indirectly promotes soil microbial activity and improves the soil microecological environment by enhancing the quantity and diversity of plant root exudates [15,60]. In Cd-contaminated soils, Brs may reduce the bioavailability of Cd, thereby decreasing their toxicity to soil microorganisms and protecting microbial community diversity [46]. However, in this study, the application of Brs did reduce the availability of soil Cd compared with the initial soil (Figure 5A). Compared to treatments without Brs application, the bioavailable fraction of soil Cd significantly increased. Therefore, from a dialectical perspective, the impact of Brs on soil ecological health depends on its degree of activation of soil Cd. Based on the aforementioned findings, the application of Brs in this study has reduced the availability of Cd compared to the initial soil conditions, which is conducive to the restoration of soil health. However, further validation through long-term research is necessary. The economic viability of applying Brs is a pivotal factor influencing the sustainable implementation of this method. Based on the purchase price of Brs in this experiment, which is approximately 0.63 USD per 100 mL, and according to the product manual, 100 mL of Brs can be diluted with water and sprayed over approximately 2668 m^2^ of wheat (Supplemental Materials). Therefore, from a cost perspective, the application of Brs to enhance phytoremediation capacity is economically feasible.

## 5. Conclusions

After four months, willow, alfalfa, and their combination effectively passivated soil Cd by elevating soil pH and CAB while reducing OPR and TOA. In contrast, Brs spray activated soil Cd by lowering soil pH and increasing TOA. However, it is noteworthy that, in comparison to soil without Brs, the concentrations of water-extracted Cd were lower in the NWABrs, likely due to increases in FMO-Cd and OM-Cd, as well as enhanced plant accumulation. Willow (NJU513) has been identified as a potential Cd hyperaccumulator, demonstrating a robust capacity to accumulate Cd in its leaves (ranging from 141.83 to 511.23 mg kg^−1^) without exhibiting toxicity responses. Furthermore, the maximum BCF of Cd in willow leaves reached 52.38. Importantly, Brs spray significantly enhanced Cd extraction by increasing the total biomass of willow and alfalfa. Additionally, Brs spray improved the resistance of willow and alfalfa to Cd by augmenting *P_n_*, photosynthetic pigments, T-SOD), total thiol (T-SH, and CAT activities, resulting in MDA levels that showed no significant negative changes compared to the CK. Overall, Brs spray enhanced the phytoremediation capabilities of alfalfa and willow towards Cd.

## Figures and Tables

**Table 1:** The physicochemical properties in the control soil (CK) and Cd-polluted soil (NWABrs), including Cd (mg kg^−1^), pH, CAB (%), oxidation-reduction potential (OPR, mV), electrical conductivity (EC, μS cm^−1^), OM (%), total organic acids (TOA, mg kg^−1^), total nickel (Ni, mg kg^−1^), total zinc (Zn, mg kg^−1^), total copper (Cu, mg kg^−1^), total nitrogen (N, mg kg^−1^), total phosphorus (P, mg kg^−1^), and total potassium (K, mg kg^−1^).

Soils	Cd	pH	CAB	OPR	EC	OM	TOA	Ni	Zn	Cu	N	P	K
CK	0.67	6.78	0.33	290	626	3.00	5.13	31	52	28	23	31	72
NWABrs	9.65	6.85	0.64	335	250	3.32	5.66	41	62	30	34	47	110

Notes: The soil NWABrs represents the initial soil of treatments of W-NBrs, A-NBrs, WA-NBrs, W-Brs, A-Brs, and WA-Brs.

**Table 2:** The specific treatment configurations and their corresponding numbers.

Treatments	CK1	CK2	CK3	NWABrs	W-NBrs	A-NBrs	WA-NBrs	W-Brs	A-Brs	WA-Brs
Plants	Willow	--	Willow	--	Willow	--	Willow	Willow	--	Willow
or	--	Alfalfa	Alfalfa	--	--	Alfalfa	Alfalfa	--	Alfalfa	Alfalfa
Brs	--	--	--	--	--	--	--	--	--	Brs

Notes: CK1, CK2, and CK3 indicate the soil with 0.67 mg kg^−1^ Cd; NWABrs, W-NBrs, A-NBrs, WA-NBrs, W-Brs, A-Brs, and WA-Brs indicate the soil with 9.65 mg kg^−1^ Cd; “--” indicates treatments without plants or Brs.

**Table 3:** Soil pH, oxidation-reduction potential (ORP), organic matter (OM), electrical conductivity (EC), total organic acid (TOA), and carbonates (CAB) after 120 days.

Treatments	pH	ORP (mv)	OM (%)	EC (μS cm^−1^)	TOA (mg kg^−1^)	CAB (%)
NWABrs	6.81 ± 0.07 ^c^	331 ± 17 ^a^	3.49 ± 0.06 ^a^	232 ± 26 ^b^	8.05 ± 1.77 ^a^	0.63 ± 0.00 ^b^
W-NBrs	7.14 ± 0.08 ^a^	205 ± 3 ^b^	3.14 ± 0.19 ^b^	616 ± 80 ^a^	4.50 ± 0.93 ^bc^	0.68 ± 0.04 ^ab^
A-NBrs	7.28 ± 0.11 ^a^	164 ± 19 ^c^	3.22 ± 0.10 ^b^	504 ± 142 ^a^	3.85 ± 1.15 ^c^	0.78 ± 0.09 ^a^
WA-NBrs	6.98 ± 0.12 ^b^	163 ± 2 ^c^	3.10 ± 0.10 ^b^	515 ± 37 ^a^	5.22 ± 0.58 ^bc^	0.72 ± 0.02 ^ab^
W-Brs	6.84 ± 0.08 ^bc^	119 ± 42 ^d^	3.23 ± 0.14 ^b^	573 ± 133 ^a^	4.91 ± 0.81 ^bc^	0.75 ± 0.04 ^ab^
A-Brs	6.73 ± 0.15 ^c^	144 ± 7 ^c^	3.13 ± 0.19 ^b^	563 ± 85 ^a^	6.61 ± 2.16 ^ab^	0.68 ± 0.09 ^ab^
WA-Brs	6.79 ± 0.07 ^c^	156 ± 8 ^c^	3.15 ± 0.09 ^b^	541 ± 49 ^a^	6.46 ± 0.26 ^bc^	0.71 ± 0.15 ^ab^

NWABrs indicates that willow and alfalfa are not planted in soil. W-NBrs, A-NBrs, and WA-NBrs indicate that soils are planted with willow, alfalfa, and a combination of willow and alfalfa, respectively. W-Brs, A-Brs, and WA-Brs indicate that willow, alfalfa, and the combination of willow and alfalfa are treated with a Brs spray. Different letters indicate significant differences among different treatments (*p* < 0.05).

## Data Availability

No new data were created or analyzed in this study.

## Supplementary Materials

The following supporting information can be downloaded at: https://www.mdpi.com/article/10.3390/plants14050765/s1, Table S1. Concentrations of Cd (average ± S.D., mg kg^−1^) in rhizosphere and non-rhizosphere soils after 4 months; Table S2. Pearson correlations among soil physicochemical properties and fractionation of Cd in rhizosphere soils after four months; Figure S1. The growth status of willow and alfalfa under the intercropping of willow and alfalfa in treatments WA-Brs, WA-NBrs, and CK3 (from left to right); Figure S2. Concentrations of water extracted Cd in rhizosphere and non-rhizosphere soils after four months. NWABrs indicates that willow and alfalfa are not planted in soil. W-NBrs, A-NBrs and WA-NBrs indicate that soils are planted with willow, alfalfa and the combination of willow and alfalfa, respectively. W-Brs, A-Brs and WA-Brs indicate that willow, alfalfa and the combination of willow and alfalfa are treated with a Brs spray. Different letters indicate significant difference among different treatments (*p* < 0.05); Figure S3. Concentrations of Cd in the leaves of willow after five months. The leaves randomly come from six willow plants in an undergoing experiment (soil Cd, 9.65 mg kg^−1^); Figure S4. Bioaccumulation factor of Cd in the leaves of willow after five months. The leaves randomly come from six willow plants in an undergoing experiment (soil Cd, 9.65 mg kg^−1^); Figure S5. 24-Epibrassinolide Instruction Manual.
