# Supplementary material for: 24-Epibrassinolide Enhanced Plant Antioxidant System and Cadmium Bioavailability Under Soil Cadmium Stress
Source: Plants (Basel). 2025 Mar 2;14(5):765. doi: 10.3390/plants14050765 (PMC11902201; doi:10.3390/plants14050765)
Supplement: Supplementary file 1 [file plants-14-00765-s001.zip › plants-3426928-supplementary.pdf]

Supplemental Materials

Table S1

Concentrations of Cd (average±S.D., mg kg^{-1}) in rhizosphere and non-rhizosphere soils after 4 months.

Treatments	NWABrs	W-NBrs	A-NBrs	WA-NBrs	W-Brs	A-Brs	WA-Brs
Rhizosphere	9.75±0.19	6.62±0.21	8.17±0.41	8.71±0.51	6.94±0.19	7.95±0.08	7.97±0.43
Non-rhizosphere		9.20±0.12	9.38±0.24	9.47±0.34	9.46±0.39	9.26±0.55	8.46±0.28

Table S2

Pearson correlations among soil physicochemical properties and fractionation of Cd in rhizosphere soils after four months.

	EX-Cd	CAB-Cd	FMO-Cd	OM-Cd	RES-Cd	pH	OPR	EC	OM	CAB	TOA
EX-Cd	1										
CAB-Cd	0.427*	1									
FMO-Cd	0.290	0.511**	1								
OM-Cd	-0.046	0.269	0.455*	1							
RES-Cd	-0.075	-0.311	-0.188	-0.663**	1						
pH	0.022	0.117	0.011	-0.257	0.497**	1					
OPR	0.627**	0.405*	-0.006	-0.471*	0.229	0.011	1				
EC	-0.422*	-0.270	0.256	0.483*	-0.204	-0.027	-0.572	1			
OM	0.128	0.232	-0.259	-0.324	0.211	-0.065	0.443*	-0.341	1		
CAB	-0.328	-0.043	0.157	0.178	0.061	0.251	-0.358	-0.250	-0.090	1	
TOA	0.408*	0.218	-0.204	-0.196	-0.133	-0.548**	0.540**	-0.520**	0.535**	-0.478*	1

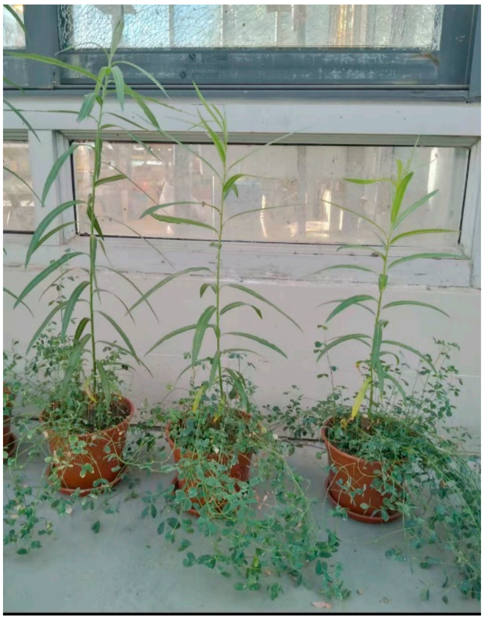

Fig. S1 The growth status of willow and alfalfa under the intercropping of willow and alfalfa in treatments WA-Brs, WA-NBrs, and CK3 (from left to right).

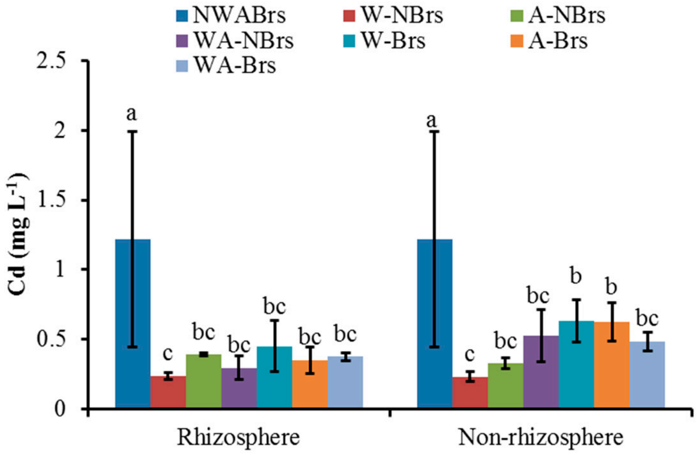

Fig.S2 Concentrations of water extracted Cd in rhizosphere and non-rhizosphere soils after four months. NWABrs indicates that willow and alfalfa are not planted in soil. W-NBrs, A-NBrs and WA-NBrs indicate that soils are planted with willow, alfalfa and the combination of willow and alfalfa, respectively. W-Brs, A-Brs and WA-Brs indicate that willow, alfalfa and the combination of willow and alfalfa are treated with a Brs spray. Different letters indicate significant difference among different treatments (p<0.05).

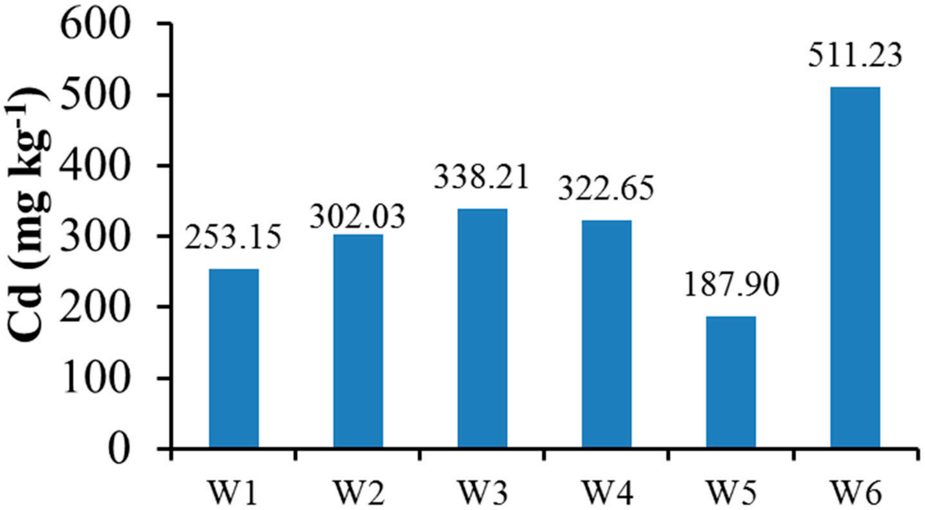

Fig.S3 Concentrations of Cd in the leaves of willow after five months. The leaves randomly come from six willow plants in an undergoing experiment (soil Cd, 9.65 mg kg^{-1}).

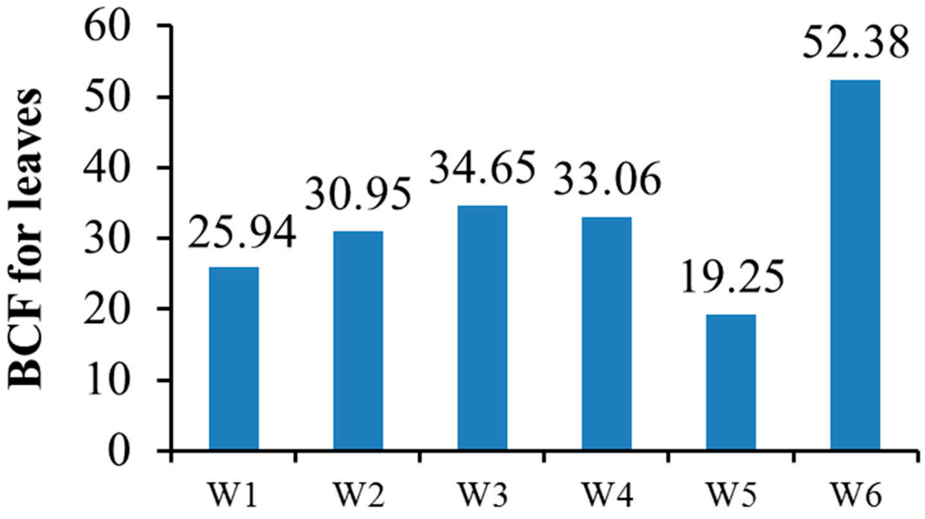

Fig.S4 Bioaccumulation factor of Cd in the leaves of willow after five months. The leaves randomly come from six willow plants in an undergoing experiment (soil Cd, 9.65 mg kg^{-1}).

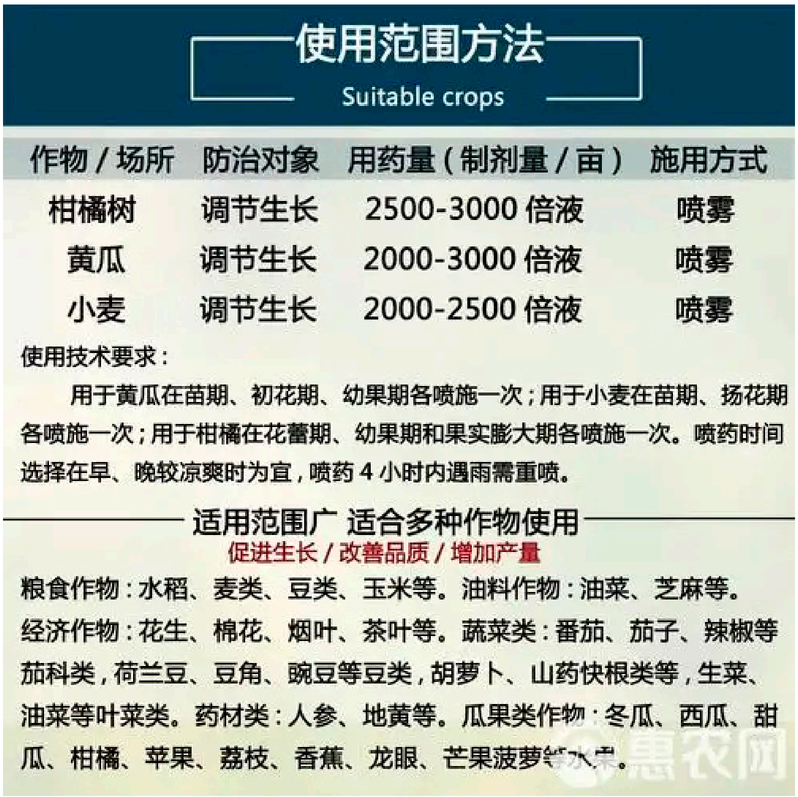

使用范围方法
Suitable crops

作物 / 场所	防治对象	用药量（制剂量 / 亩）	施用方式
柑橘树	调节生长	2500-3000 倍液	喷雾
黄瓜	调节生长	2000-3000 倍液	喷雾
小麦	调节生长	2000-2500 倍液	喷雾

使用技术要求：

用于黄瓜在苗期、初花期、幼果期各喷施一次；用于小麦在苗期、扬花期各喷施一次；用于柑橘在花蕾期、幼果期和果实膨大期各喷施一次。喷药时间选择在早、晚较凉爽时为宜，喷药 4 小时内遇雨需重喷。

适用范围广 适合多种作物使用

促进生长 / 改善品质 / 增加产量

粮食作物：水稻、麦类、豆类、玉米等。油料作物：油菜、芝麻等。经济作物：花生、棉花、烟叶、茶叶等。蔬菜类：番茄、茄子、辣椒等茄科类，荷兰豆、豆角、豌豆等豆类，胡萝卜、山药快根类等，生菜、油菜等叶菜类。药材类：人参、地黄等。瓜果类作物：冬瓜、西瓜、甜瓜、柑橘、苹果、荔枝、香蕉、龙眼、芒果菠萝等水果。

惠农网

Fig.S5 24-Epibrassinolide Instruction Manual (Chinese version)

The method of using scope			
Crop	Function	Dosage (per treatment dose/acre)	Application method
Orange trees	Regulation growth	2500-3000 times liquid	Spray
Cucumbers	Regulation growth	2000-3000 times liquid	Spray
wheat	Regulation growth	2000-2500 times liquid	Spray
Cucumbers: Spray once each during the seedling stage, initial flowering stage, and young fruit stage.<br>Wheat: Spray once each during the seedling stage and flowering (anthesis) stage.<br>Citrus: Spray once each during the bud stage, young fruit stage, and fruit expansion stage.<br>Timing: Apply in the early morning or late evening when temperatures are cooler.<br>If rainfall occurs within 4 hours after application, re-spray is required.			
Suitable Crops			
**Food Crops:** Rice, wheat, legumes, corn, etc.<br>**Oil Crops:** Rapeseed, sesame, etc.<br>**Cash Crops:** Peanuts, cotton, tobacco, tea, etc.<br>**Vegetables:** Solanaceae: Tomato, eggplant, pepper, etc.<br>**Legumes:** Snow peas, cowpeas, peas, etc.<br>**Root Vegetables:** Carrot, yam, etc.<br>**Leafy Greens:** Lettuce, rapeseed greens, etc.<br>**Medicinal Plants:** Ginseng, rehmannia, etc.<br>**Fruit and Melon Crops:**<br>Melons: Winter melon, watermelon, muskmelon, etc.<br>Fruits: Citrus, apple, lychee, banana, longan, mango, pineapple, etc.			

Fig.S5 24-Epibrassinolide Instruction Manual (English version)
